# Seminator 2 Can Complement Generalized Büchi Automata via Improved Semi-determinization

**DOI:** 10.1007/978-3-030-53291-8_2

**Published:** 2020-06-16

**Authors:** František Blahoudek, Alexandre Duret-Lutz, Jan Strejček

**Affiliations:** 8 Microsoft Research Lab, Redmond, WA USA; 9 University of Southern California, Los Angeles, CA USA; 10 University of Texas at Austin, Austin, USA; 11 LRDE, EPITA, Le Kremlin-Bicêtre, France; 12 Masaryk University, Brno, Czech Republic

## Abstract

We present the second generation of the tool Seminator that transforms transition-based generalized Büchi automata (TGBAs) into equivalent semi-deterministic automata. The tool has been extended with numerous optimizations and produces considerably smaller automata than its first version. In connection with the state-of-the-art LTL to TGBAs translator Spot, Seminator  2 produces smaller (on average) semi-deterministic automata than the direct LTL to semi-deterministic automata translator ltl2ldgba of the Owl library. Further, Seminator  2 has been extended with an improved NCSB complementation procedure for semi-deterministic automata, providing a new way to complement automata that is competitive with state-of-the-art complementation tools.

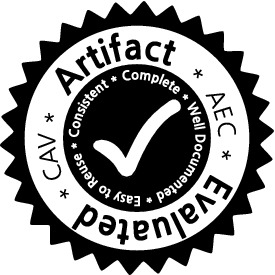



## Introduction

*Semi-deterministic* [24] automata are automata where each accepting run makes only finitely many nondeterministic choices. The merit of this interstage between deterministic and nondeterministic automata comes from two facts known since the late 1980s. First, every nondeterministic Büchi automaton with *n* states can be transformed into an equivalent semi-deterministic Büchi automaton with at most $4^n$ states [7, 24]. Note that asymptotically optimal determinization procedures transform nondeterministic Büchi automata to deterministic automata with $2^{\mathcal {O}(n\log n)}$ states [24] and with a more complex (typically Rabin) acceptance condition, as deterministic Büchi automata are strictly less expressive. Second, some algorithms cannot handle nondeterministic automata, but they can handle semi-deterministic ones; for example, algorithms for qualitative model checking of *Markov decision processes* (MDPs) [7, 29].

For theoreticians, the difference between the complexity of determinization and semi-determinization is not dramatic—both constructions are exponential. However, the difference is important for authors and users of practical automata-based tools—automata size and the complexity of their acceptance condition often have a significant impact on tool performance. This latter perspective has recently initiated another wave of research on semi-deterministic automata. Since 2015, many new results have been published: several direct translations of LTL to semi-deterministic automata [11, 15, 16, 26], specialized complementation constructions for semi-deterministic automata [4, 6], algorithms for quantitative model checking of MDPs based on semi-deterministic automata [13, 25], a transformation of semi-deterministic automata to deterministic parity automata [10], and reinforcement learning of control policy using semi-deterministic automata [21].

In 2017, we introduced Seminator  1.1 [5], a tool that transforms nondeterministic automata to semi-deterministic ones. The original semi-determinization procedure of Courcoubetis and Yannakakis [7] works with standard *Büchi automata* (BAs). Seminator  1.1 extends this construction to handle more compact automata, namely *transition-based Büchi automata* (TBAs) and *transition-based generalized Büchi automata* (TGBAs). TBAs use accepting transitions instead of accepting states, and TGBAs have several sets of accepting transitions, each of these sets must be visited infinitely often by accepting runs. The main novelty of Seminator  1.1 was that it performed degeneralization and semi-determinization of a TGBA simultaneously. As a result, it could translate TGBAs to smaller semi-deterministic automata than (to our best knowledge) the only other tool for automata semi-determinization called nba2ldba [26]. This tool only accepts BAs as input, and thus TGBAs must be degeneralized before nba2ldba is called.

Moreover, in connection with the LTL to TGBAs translator ltl2tgba of Spot [8], Seminator  1.1 provided a translation of LTL to semi-deterministic automata that can compete with the direct LTL to semi-deterministic TGBAs translator ltl2ldba [26]. More precisely, our experiments [5] showed that the combination of ltl2tgba and Seminator  1.1 outperforms ltl2ldba on LTL formulas that ltl2tgba translates directly to deterministic or semi-deterministic TGBA (i.e., when Seminator has no work to do), while ltl2ldba produced (on average) smaller semi-deterministic TGBAs on the remaining LTL formulas (i.e., when the TGBA produced by ltl2tgba has to be semi-determinized by Seminator).

This paper presents Seminator  2, which changes the situation. With many improvements in semi-determinization, the combination of ltl2tgba and Seminator  2 now translates LTL to smaller (on average) semi-deterministic TGBAs than ltl2ldba even for the cases when ltl2tgba produces a TGBA that is not semi-deterministic. Moreover, this holds even when we compare to ltl2ldgba, which is the current successor of ltl2ldba distributed with Owl [19].

Further, Seminator  2 now provides a new feature: *complementation of TGBAs*. Seminator  2 chains semi-determinization with the complementation algorithm called NCSB [4, 6], which is tailored for semi-deterministic BAs. Our experiments show that the complementation in Seminator  2 is fully competitive with complementations implemented in state-of-the-art tools [1, 8, 20, 23, 30].

## Improvements in Semi-determinization

First of all, we recall the definition of semi-deterministic automata and principles of the semi-determinization procedure implemented in Seminator  1.1 [5].

Let $\mathcal {A}=(Q,\varSigma ,\delta ,q_0,\{F_1,\ldots ,F_n\})$ be a TGBA over alphabet $\varSigma$, with a finite set of states *Q*, a transition relation $\delta \subseteq Q\times \varSigma \times Q$, an initial state $q_0\in Q$, and sets of accepting transitions $F_1,\ldots ,F_n\subseteq \delta$. Then $\mathcal {A}$ is *semi-deterministic* if there exists a subset $Q_D\subseteq Q$ such that (i) each transition from $Q_D$ goes back to $Q_D$ (i.e., $\delta \cap (Q_D\times \varSigma \times (Q\smallsetminus Q_D))=\emptyset$), (ii) all states of $Q_D$ are deterministic (i.e., for each $q\in Q_D$ and $a\in \varSigma$ there is at most one $q'$ such that $(q,a,q')\in \delta$), and (iii) each accepting transition starts in a state of $Q_D$ (i.e., $F_1,\ldots ,F_n\subseteq Q_D\times \varSigma \times Q_D$).

**Fig. 1.**
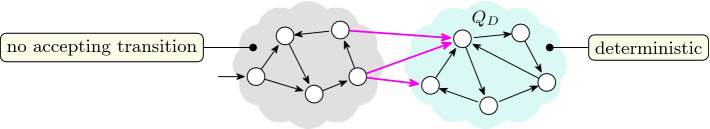
Structure of a semi-deterministic automaton. The deterministic part contains all accepting transitions and states reachable from them. Cut-transitions are magenta.

The part of $\mathcal {A}$ delimited by states of $Q_D$ is called *deterministic*, while the part formed by the remaining states $Q\smallsetminus Q_D$ is called *nondeterministic*, although it could contain deterministic states too. The transitions leading from the nondeterministic part to the deterministic one are called *cut-transitions*. The structure of a semi-deterministic automaton is depicted in Fig. 1.

Intuitively, a TGBA $\mathcal {A}$ with a set of states *Q* and a single set of accepting transitions *F* can be transformed into a semi-deterministic TBA $\mathcal {B}$ as follows. First, we use a copy of $\mathcal {A}$ as the nondeterministic part of $\mathcal {B}$. The deterministic part of $\mathcal {B}$ has states of the form (*M*, *N*) such that $Q\supseteq M\supseteq N$ and $M\ne \emptyset$. Every accepting transition $(q,a,q')\in F$ induces a cut-transition $(q,a,(\{q'\},\emptyset ))$ of $\mathcal {B}$. The deterministic part is then constructed to track all runs of $\mathcal {A}$ from each such state $q'$ using the powerset construction. More precisely, the first element of (*M*, *N*) tracks all runs while the second element tracks only the runs that passed some accepting transition of *F*. Each transition of the deterministic part, that would reach a state where $M=N$ (so-called *breakpoint*) is replaced with an accepting transition of $\mathcal {B}$ leading to state $(M,N')$, where $N'$ tracks only the runs of $\mathcal {A}$ passing an accepting transition of *F* in the current step.

Seminator  1.1 extended this procedure to construct a semi-deterministic TBA even for a TGBA with multiple acceptance sets $F_1,\ldots ,F_n$. States of the deterministic part are now triples (*M*, *N*, *i*), where $i\in \{0,\ldots ,n-1\}$ is called *level* and it has a similar semantics as in degeneralization. Cut-transitions are induced by transitions of $F_n$ and they lead to states of the form $(\{q'\},\emptyset ,0)$. The level *i* says that *N* tracks runs that passed a transition of $F_{i+1}$ since the last level change. When the deterministic part reaches a state (*M*, *N*, *i*) with $M=N$, we change the level to $i'=(i+1)\bmod n$ and modify *N* to track only runs passing $F_{i'+1}$ in the current step. Transitions changing the level are accepting.

A precise description of these semi-determinization procedures and proofs of their correctness can be found in Blahoudek’s dissertation [3]. Now we briefly explain the most important optimizations added in Seminator  2 (we work on a journal paper with their formal description). Each optimization can be enabled/disabled by the corresponding option. All of them are enabled by default.

--scc-aware approach identifies, for each cut-transition, the strongly connected component (SCC) of $\mathcal {A}$ that contains the target of the transition triggering the cut-transition. The sets *M*, *N* then track only runs staying in this SCC.
--reuse-deterministic treats in a specific way each deterministic SCC from which only deterministic SCCs are reachable in $\mathcal {A}$: it (i) does not include them in the nondeterministic part, and (ii) copies them (and their successors) in the deterministic part as they are, including the original acceptance transitions. This optimization can result in a semi-deterministic TGBA with multiple acceptance sets on output.
--cut-always changes the policy *when* cut-transitions are created: they are now triggered by all transitions of $\mathcal {A}$ with the target state in an accepting SCC.
--powerset-on-cut applies the powerset construction when computing targets of cut-transitions. The target of a cut-transition leading from *q* is constructed in the same way as the successor of the hypothetical state $(\{q\},\emptyset ,0)$ of the deterministic part.
--skip-levels is a variant of the level jumping trick from TGBA degeneralization [2]. Roughly speaking, a single transition in the deterministic part can change the level *i* directly to $i+j$ where $j\ge 1$ if all runs passed acceptance transitions from all the sets $F_{i+1},\ldots ,F_{i+j}$ in the current step.
--jump-to-bottommost makes sure that all cut-transitions leading to states with the same *M* component lead to the same state (*M*, *N*, *i*) for some *N* and *i*. It relies on the fact that each run takes only one cut-transition, and thus only the component *M* of the cut-transition’s target state is important for determining the acceptance of the run. During the original construction, many states of the form $(M,N',i')$ may appear in different SCCs. After the construction finishes, this optimization redirects each cut-transition leading to $(M,N',i')$ to some state of the form (*M*, *N*, *i*) that belongs to the bottommost SCC (in a topological ordering of the SCCs) that contains such a state. This is inspired by a similar trick used by Křetínský et al. [18] in a different context.
--powerset-for-weak simplifies the construction for weak accepting SCCs (i.e., SCCs where all cycles are accepting) of $\mathcal {A}$. For such SCCs it just applies the powerset construction (builds states of the form *M* instead of triples (*M*, *N*, *i*)) with all transitions accepting in the deterministic part.

Note that Seminator  1.1 can produce a semi-deterministic TGBA with multiple acceptance sets only when it gets a semi-deterministic TGBA as input. Seminator  2 produces such automata more often due to --reuse-deterministic.

## Implementation and Usage

Seminator 2 is an almost complete rewrite of Seminator [5], and is still distributed under the GNU GPL 3.0 license. Its distribution tarball and source code history are hosted on GitHub (https://github.com/mklokocka/seminator). The package contains sources of the tool with two user-interfaces (a command-line tool and Python bindings), a test-suite, and some documentation.

Seminator is implemented in C++ on top of the data-structures provided by the Spot library [8], and reuses its input/output functions, simplification algorithms, and the NCSB complementation. The main implementation effort lies in the optimized semi-determinization and an alternative version of NCSB.

**Fig. 2.**
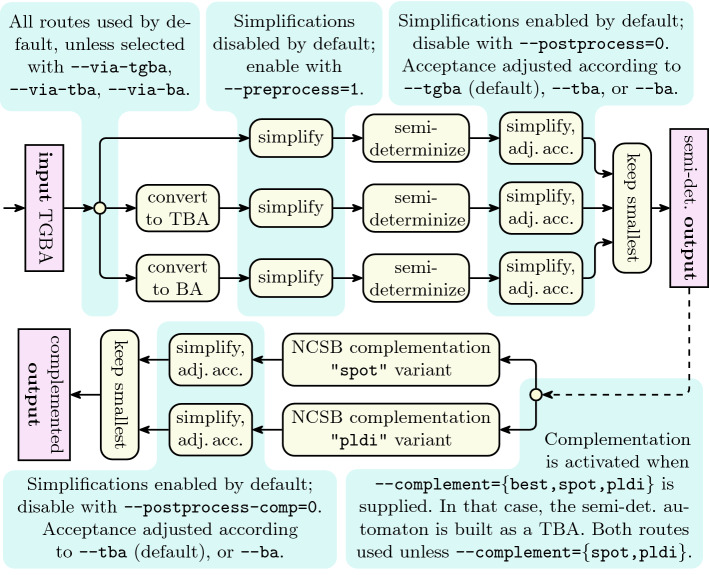
Workflow for the two operation modes of seminator: semi-determinizing and complementing via semi-determinization.

The first user interface is a command-line tool called seminator. Its high-level workflow is pictured in Fig. 2. By default (top-part of Fig. 2) it takes a TGBA (or TBA or BA) on input and produces a semi-deterministic TGBA (or TBA or BA if requested). Figure 2 details various switches that control the optional simplifications and acceptance transformations that occur before the semi-determinization itself. The pre- and post-processing are provided by the Spot library. The semi-determinization algorithm can be adjusted by additional command-line options (not shown in Fig. 2) that enable or disable optimizations of Sect. 2. As Spot simplification routines are stronger on automata with simpler acceptance conditions, it sometimes pays off to convert the automaton to TBA or BA first. If the input is a TGBA, seminator attempts three semi-determinizations, one on the input TGBA, one on its TBA equivalent, and one on its BA equivalent; only the smallest result is retained. If the input is already a TBA (resp. a BA), only the last two (resp. one) routes are attempted.

The --complement option activates the bottom part of Fig. 2 with two variants of the NCSB complementation [4]: "spot" stands for a transition-based adaptation of the original algorithm (implemented in Spot); "pldi" refers to its modification based on the optimization by Chen et al. [6, Section 5.3] (implemented in Seminator  2). Both variants take a TBA as input and produce a TBA. The options --tba and –ba apply on the final complement automaton only.

The seminator tool can now process automata in batch, making it possible to build pipelines with other commands. For instance the pipeline



uses Spot’s ltl2tgba command to read a list of LTL formulas from input.ltl and transform it into a stream of TGBAs that is passed to seminator, which transforms them into semi-deterministic TGBAs, and finally Spot’s autfilt saves into output.hoa the automata with 3 states or more.

Python bindings form the second user-interface and are installed by the Seminator package as an extension of Spot’s own Python bindings. It offers several functions, all working with Spot’s automata (twa_graph objects):

- semi_determinize() implements the semi-determinization procedure;
- complement_semidet() implements the "pldi" variant of the NCSB complementation for semi-deterministic automata (the other variant is available under the same function name in the bindings of Spot);
- highlight_components() and highlight_cut() provide ways to highlight the nondeterministic and the deterministic parts of a semi-deterministic automaton, and its cut-transitions;
- seminator() provides an interface similar to the command-line seminator tool with options that selectively enable or disable optimizations or trigger complementation.

**Fig. 3.**
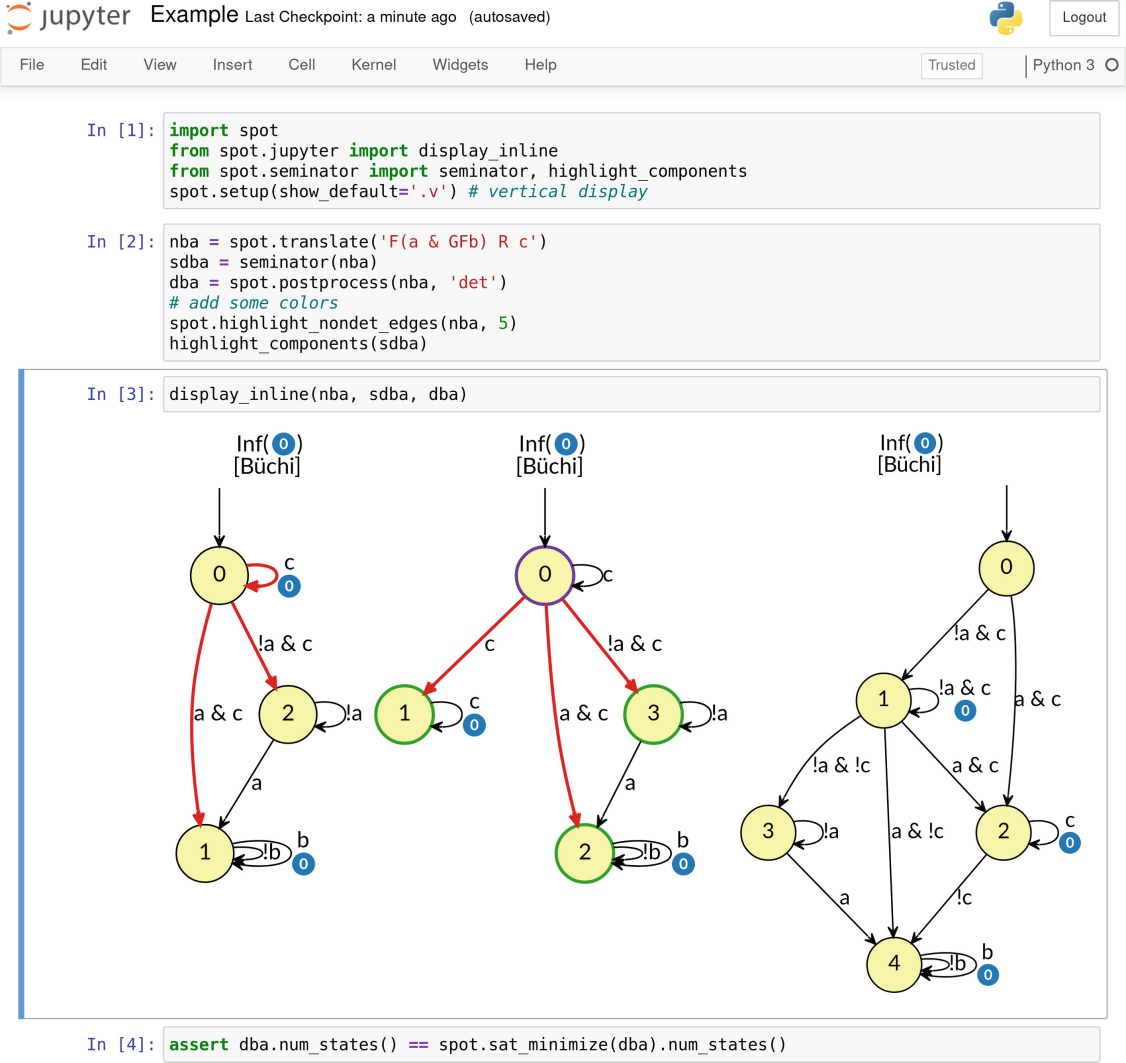
Jupyter notebook illustrating a case where a nondeterministic TBA (nba, left) has an equivalent semi-deterministic TBA (sdba, middle) that is smaller than a minimal deterministic TBA (dba, right). Accepting transitions are labeled by

The Python bindings integrate well with the interactive notebooks of Jupyter [17]. Figure 3 shows an example of such a notebook, using the seminator() and highlight_components() functions. Additional Jupyter notebooks, distributed with the tool, document the effect of the various optimization options.^1^

## Experimental Evaluation

We evaluate the performance of Seminator  2 for both semi-determinization and complementation of TGBAs. We compare our tool against several tools listed in Table 1. As ltl2ldgba needs LTL on input, we used the set of 221 LTL formulas already considered for benchmarking in the literature [9, 12, 14, 22, 27]. To provide TGBAs as input for Seminator 2, we use Spot’s ltl2tgba to convert the LTL formulas. Based on the automata produced by ltl2tgba, we distinguish three categories of formulas: *deterministic* (152 formulas), *semi-deterministic* but not deterministic (49 formulas), and *not semi-deterministic* (20 formulas). This division is motivated by the fact that Seminator  2 applies its semi-determinization only on automata that are not semi-deterministic, and that some complementation tools use different approaches to deterministic automata. We have also generated 500 random LTL formulas of each category.

The scripts and formulas used in those experiments can be found online,^2^ as well as a Docker image with these scripts and all the tools installed.^3^ All experiments were run inside the supplied Docker image on a laptop Dell XPS13 with Intel i7-1065G7, 16 GB RAM, and running Linux.

**Table 1.** Versions and references to the other tools used in our evaluation.

Package (Tool)	Version	Ref.
Fribourg plugin for GOAL	(na)	[1, 30]
GOAL (gc)	20200506	[28]
Owl (ltl2ldgba)	19.06.03	[11]
ROLL (replaces Buechic)	1.0	[20]
Seminator (seminator)	1.1	[5]
Spot (autfilt, ltl2tgba)	2.9	[8]

**Fig. 4.**
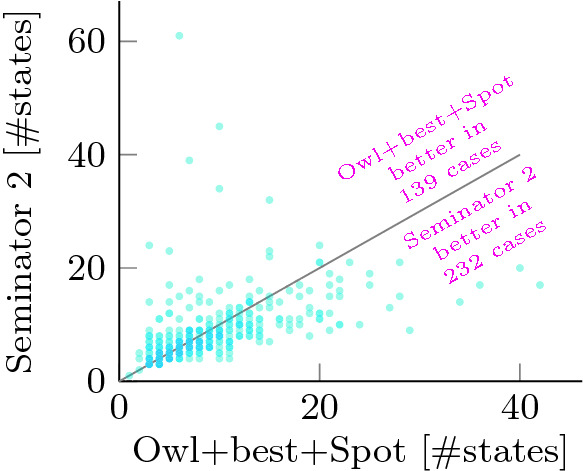
Comparison of the sizes of the semi-deterministic automata produced by Seminator 2 and Owl for the *not semi-deterministic* random set.

**Table 2.**
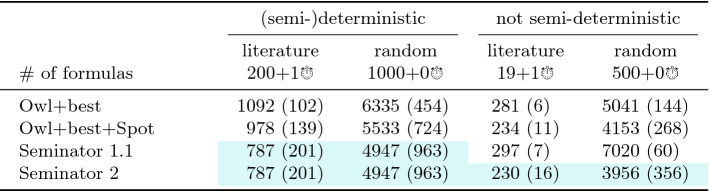
Comparison of semi-determinization tools. A benchmark set marked with $x + y$  consists of *x* formulas for which all tools produced some automaton, and *y* formulas leading to some timeouts. A cell of the form $s\,(m)$ shows the cumulative number *s* of states of automata produced for the *x* formulas, and the number *m* of formulas for which the tool produced the smallest automaton out of the obtained automata. The best results in each column are highlighted.

### Semi-determinization

We compare Seminator 2 to its older version 1.1 and to ltl2ldgba of Owl. We do not include Buchifier [16] as it is available only as a binary for Windows. Also, we did not include nba2ldba [26] due to the lack of space and the fact that even Seminator 1.1 performs significantly better than nba2ldba [5].

Recall that Seminator  2 calls Spot’s automata simplification routines on constructed automata. To get a fair comparison, we apply these routines also to the results of other tools, indicated by *+Spot* in the results. Further, ltl2ldgba of Owl can operate in two modes: --symmetric and --asymmetric. For each formula, we run both settings and pick the better result, indicated by *+best*.

Table 2 presents the cumulative results for each semi-determinization tool and each benchmark set (we actually merged *deterministic* and *semi-deterministic* benchmark sets). The timeout of 30 s was reached by Owl for one formula in the *(semi-)deterministic* category and by Seminator  1.1 for one formula in the *not semi-deterministic* category. Besides timeouts, the running times of all tools were always below 3 s, with a few exceptions for Seminator  1.1.

In the *(semi-)deterministic* category, the automaton produced by ltl2tgba and passed to both versions of Seminator is already semi-deterministic. Hence, both versions of Seminator have nothing to do. This category, in fact, compares ltl2tgba of Spot against ltl2ldgba of Owl.

Figure 4 shows the distribution of differences between semi-deterministic automata produced by Owl+best+Spot and Seminator  2 for the *not semi-deterministic* random set. A dot at coordinates (*x*, *y*) represents a formula for which Owl and Seminator 2 produced automata with *x* and *y* states, respectively.

We can observe a huge improvement brought by Seminator 2 in *not semi-deterministic* benchmarks: while in 2017 Seminator 1.1 produced a smaller automaton than Owl in only few cases in this category [5], Seminator 2 is now more than competitive despite the fact that also Owl was improved over the time.

### Complementation

We compare Seminator  2 with the complementation of ROLL based on automata learning (formerly presented as Buechic), the determinization-based algorithm [23] implemented in GOAL, the asymptotically optimal Fribourg complementation implemented as a plugin for GOAL, and with Spot (autfilt --complement). We apply the simplifications from Spot to all results and we use Spot’s ltl2tgba to create the input Büchi automata for all tools, using transition-based generalized acceptance or state-based acceptance as appropriate (only Seminator 2 and Spot can complement transition-based generalized Büchi automata). The timeout of 120 s was reached once by both Seminator 2 and Spot, 6 times by Fribourg, and 13 times by GOAL and ROLL.

**Table 3.**
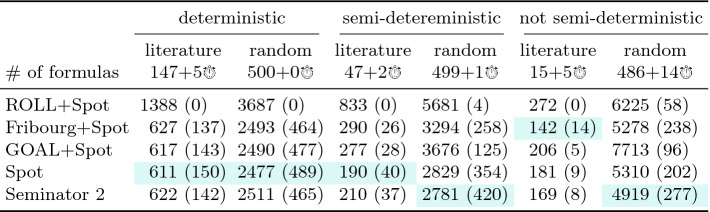
Comparison of tools complementing Büchi automata, using the same conventions as Table 2.

**Fig. 5.**
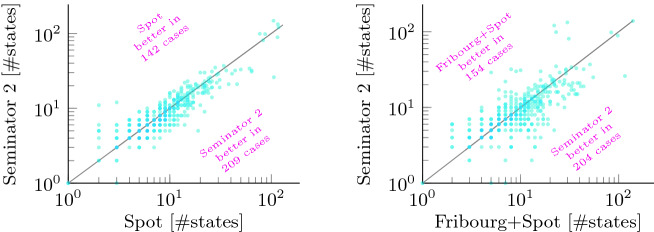
Comparison of Seminator 2 against Spot and Fribourg+Spot in terms of the sizes (i.e., number of states) of complement automata produced for the *not semi-deterministic* random benchmark. Note that axes are logarithmic.

Table 3 shows results for complementation in the same way as Table 2 does for semi-determinization. For the *deterministic* benchmark, we can see quite similar results from all tools but ROLL. This is caused by the fact that complementation of deterministic automata is easy. Some tools (including Spot) even apply a dedicated complementation procedure. It comes at no surprise that the specialized algorithm of Seminator  2 performs better than most other complementations in the *semi-deterministic* category. Interestingly, this carries over to the *not semi-deterministic* category. The results demonstrate that the 2-step approach of Seminator  2 to complementation performs well in practice. Figure 5 offers more detailed insight into distribution of automata sizes created by Seminator  2, Spot, and Fribourg+Spot for random benchmarks in this category.

**Fig. 6.**
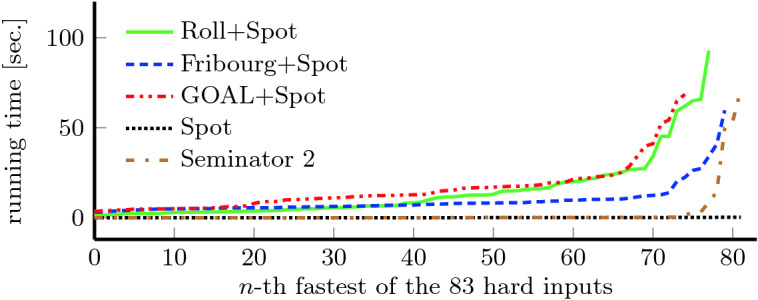
Running times of complementation tools on the 83 hard cases of the *not semi-deterministic* random benchmark. The running times of each tool on these cases are sorted increasingly before being plotted.

Finally, Fig. 6 compares the running times of these tools over the 83 hard cases of *not semi-deterministic* random benchmark (a case is *hard* if at least one tool did not finish in 10 s). We can see that Seminator  2 and Spot run significantly faster than the other tools.

## Conclusion

We have presented Seminator  2, which is a substantially improved version of Seminator  1.1. The tool now offers a competitive complementation of TGBA. Furthermore, the semi-determinization code was rewritten and offers new optimizations that significantly reduce the size of produced automata. Finally, new user-interfaces enable convenient processing of large automata sets thanks to the support of pipelines and batch processing, and versatile applicability in education and research thanks to the integration with Spot’s Python bindings.

## Footnotes

1 https://nbviewer.jupyter.org/github/mklokocka/seminator/tree/v2.0/notebooks/.

2 https://github.com/xblahoud/seminator-evaluation/.

3 https://hub.docker.com/r/gadl/seminator.
